# Altered high-density lipoprotein composition and functions during severe COVID-19

**DOI:** 10.1038/s41598-021-81638-1

**Published:** 2021-01-27

**Authors:** Floran Begue, Sébastien Tanaka, Zarouki Mouktadi, Philippe Rondeau, Bryan Veeren, Nicolas Diotel, Alexy Tran-Dinh, Tiphaine Robert, Erick Vélia, Patrick Mavingui, Marie Lagrange-Xélot, Philippe Montravers, David Couret, Olivier Meilhac

**Affiliations:** 1 INSERM, UMR 1188 Diabète atherothombose Réunion Océan Indien (DéTROI), Université de La Réunion, Saint-Denis de La Réunion, France; 2 AP-HP, Service d’Anesthésie-Réanimation, CHU Bichat-Claude Bernard, 75018 Paris, France; 3 Université de Paris, UFR Denis Diderot, Paris, France; 4 AP-HP, Service de Biochimie, CHU Bichat-Claude Bernard, 75018 Paris, France; 5 Clinique Sainte-Clotilde, Groupe Clinifutur, Pôle mère enfant, 97490 Sainte-Clotilde, La Réunion, France; 6 UMR Processus Infectieux en Milieu Insulaire Tropical (PIMIT), INSERM 1187, CNRS 9192, IRD 249, Université de La Réunion, 2 rue Maxime Rivière (GIP CYROI), 97490 Sainte-Clotilde, La Réunion, France; 7 Service des maladies infectieuses, CHU de La Réunion, Saint-Denis, France; 8 Inserm UMR 1152 Physiopathologie Et Épidémiologie Des Maladies Respiratoires, Université Diderot, Paris, France; 9 Service de neuro-réanimation, CHU de La Réunion, Saint-Pierre, France; 10 CIC-EC 1410, CHU de La Réunion, Saint-Pierre, France

**Keywords:** Proteomics, Biomarkers, Viral infection, Apoptosis, Mass spectrometry

## Abstract

Coronavirus disease 2019 (COVID-19) pandemic is affecting millions of patients worldwide. The consequences of initial exposure to SARS-CoV-2 go beyond pulmonary damage, with a particular impact on lipid metabolism. Decreased levels in HDL-C were reported in COVID-19 patients. Since HDL particles display antioxidant, anti-inflammatory and potential anti-infectious properties, we aimed at characterizing HDL proteome and functionality during COVID-19 relative to healthy subjects. HDLs were isolated from plasma of 8 severe COVID-19 patients sampled at admission to intensive care unit (Day 1, D1) at D3 and D7, and from 16 sex- and age-matched healthy subjects. Proteomic analysis was performed by LC-MS/MS. The relative amounts of proteins identified in HDLs were compared between COVID-19 and controls. apolipoprotein A-I and paraoxonase 1 were confirmed by Western-blot analysis to be less abundant in COVID-19 versus controls, whereas serum amyloid A and alpha-1 antitrypsin were higher. HDLs from patients were less protective in endothelial cells stiumalted by TNFα (permeability, VE-cadherin disorganization and apoptosis). In these conditions, HDL inhibition of apoptosis was blunted in COVID-19 relative to controls. In conclusion, we show major changes in HDL proteome and decreased functionality in severe COVID-19 patients.

## Introduction

Severe acute respiratory syndrome (SARS) coronavirus 2 (SARS-CoV-2) is causing a major worldwide pandemic associated with respiratory symptoms characterized by acute lung injury, and rapidly progressing to acute respiratory distress syndrome (ARDS). Lung dysfunction is rapidly paralleled by an important “cytokine storm” in which inflammatory cytokines are abundantly released into the blood stream by immune and non-immune cells, leading to host tissue damage^1^. Several studies reported important modifications in the lipid profile of COVID-19 patients. In a small cohort of patients, Fan et al*.* suggested that decreased low-density lipoprotein cholesterol (LDL-C) concentration could be a predictor of poor prognosis. In non-survivor patients (n = 4) they report that LDL-C levels decreased continuously until death and inversely correlated with high-sensitivity C- reactive protein (CRP) levels^2^. Wang et al. reported positive correlations between COVID-19 severity and several biochemical markers including serum amyloid A (SAA), CRP or procalcitonin and negative correlation with albumin and HDL-C^3^. Another study suggested that COVID-19 patients (n = 114) presented a marked decrease in both LDL-C and HDL-C, the latter being associated with the severity of the disease^4^. We recently reported that LDL-C concentrations in COVID-19 patients were associated with mortality in case of bacterial superinfection during ICU hospitalization^5^. The dramatic decrease in albumin and lipoproteins as well as the release of acute phase proteins (SAA, CRP and lactate dehydrogenase) support the hypothesis that these parameters may represent surrogate markers of hepatic dysfunction and hepatotoxicity caused by COVID-19-induced cytokine storm^6^. HDL particles display pleiotropic effects, in addition to their well-known reverse transport cholesterol function from peripheral tissues back to the liver, such as antioxidant, anti-inflammatory and even anti-infectious properties^7,8^. Beyond the quantitative aspect, nothing has been reported yet regarding the structure, composition, and functionality of HDL particles in COVID-19. Impaired HDL function may participate in endothelial loss of integrity, increased oxidative stress and inflammation which aggravates the pathophysiology of COVID-19, characterized by a severe endothelial injury associated with the presence of intracellular virus^9^. HDL particles are profoundly altered in low-grade and acute inflammation^10–12^. HDL modifications may affect their protein cargo and their lipid content, producing "pro-inflammatory HDL particles"^13,14^. These particles, in addition to losing their endothelial protective effects, may induce monocyte/macrophage production of TNFα and MCP-1^15^. Whereas modifications of HDL-C levels, particle size and function are well documented in bacterial sepsis, only scarce information is available for viral infection^12,16^. The aim of the present study was to characterize HDL protein composition and functionality in severe COVID-19. For this purpose, HDLs were isolated by ultracentrifugation from plasma of severe COVID-19 patients the day of their admission to the intensive care unit (ICU) (D1), at D3 and D7 for survivors. Shotgun proteomics was performed on HDL fractions and their endothelial protective effect was tested on HUVECs (human umbilical vein endothelial cells).

## Results

### Characteristics of the study population

The main characteristics of COVID-19 cases and controls included in the study are detailed in Table 1. No significant difference was observed regarding age, gender and BMI between COVID-19 patients and controls. Controls were matched for age and gender (2 controls /patient). Severe COVID-19 patients have a median age of 51 [46–62] years old and male represent 63% of the population. Among COVID-19 patients, 12.5% had prior high blood pressure, 12.5% had a diabetes mellitus and 12.5% use statins. At admission, SAPSII and SOFA scores were 44 [41–56] and 6 [5–7], respectively. During their ICU stay, 75% were in septic shock, 25% in sepsis; all patients required mechanical ventilation with a median length of mechanical ventilation of 11 [6–31] days. 50% of patients needed prone positioning and 25% assistance by ECMO. Renal replacement therapy was necessary for 12.5% of patients. Day-28 mortality was 37.5% whereas ICU and hospital length of stay were 16 [6–32] and 22 [10–39] days respectively.

**Table 1:** General characteristics and outcome of ICU COVID-19 patients and control subjects.

Characteristics	ICU COVID-19 (n = 8)	Controls (n = 16)
Age, years, median [IQR]	51 [46–62]	50.5 [46–57]
Male sex, n (%)	5 (63)	9 (56)
BMI, kg/m^2^, median [IQR]	29 [23–31]	23.5 [22–26]
**Presence of comorbidities**		
High blood pressure, n (%)	1 (12.5)	2 (12.5)
Diabetes mellitus, n (%)	1 (12.5)	0 (0)
Statin use, n (%)	1 (12.5)	0 (0)
**Timing of hospitalization**		
Between first symptoms and hospitalization (days)	8 [2–11]	
Between hospitalization and ICU admission (days)	3 [2–5]	
**Severity scores at admission**		
SAPSII, median [IQR]	44 [41–56]	
SOFA, median [IQR]	6 [5–7]	
**Treatments during ICU stay**		
Norepinephrine, n (%)	6 (75)	
Mechanical ventilation, n (%)	8 (100)	
Length of mechanical ventilation, median [IQR]	11 [6–31]	
Prone positioning, n (%)	4 (50)	
Tracheostomy, n (%)	1 (12.5)	
ECMO, n (%)	2 (25)	
RRT, n (%)	1 (12.5)	
**COVID specific treatments**		
Lopinavir/ritonavir, n (%)	0 (0)	
Hydroxychloroquine, n (%)	2 (25)	
Corticosteroids, n (%)	2 (25)	
**Outcome**		
ICU LOS, median [IQR]	16 [6–32]	
Hospital LOS, median [IQR]	22 [10–39]	
Mortality at day-28, n (%)	3 (37.5)	

BMI, body mass index; ECMO, extracorporeal membrane oxygenation; LOS, length of stay; RRT, renal replacement therapy; SAPS II, simplified acute physiology score II.

Lipid profiles were significantly affected by COVID-19 with decreased total cholesterol, HDL-C and LDL-C levels and increased triglyceride concentration at admission (D1) relative to control subjects (Table 2). Plasma ApoA-I quantified by mass spectrometry was decreased by 55% in patients versus controls. A good correlation was observed between ApoA-I and HDL-C levels (r = 0.94, *p* < 0.0001, Supplemental Figure 1).

**Table 2:** Lipid profile and plasma ApoA-I concentration in COVID patients and control subjects.

Lipid profile	Admission (D1)	Day 3	Day 7	Controls	*p* values (D1 vs Controls)
TC, mmol/L, median [IQR]	2.65 [2.46–3.88]	2.68 [2.30–3.24]	4.09 [3.21–4.89]	5.25 [4.83–5.73]	0.0016
TG, mmol/L, median [IQR]	2.53 [1.73–2.99]	2.73 [2.0–3.18]	2.11 [2.02–3.60]	1.20 [0.90–1.83]	0.0152
HDL-C, mmol/L, median [IQR]	0.77 [0.49–0.83]	0.58 [0.44–0.86]	0.84 [0.64–0.91]	1.46 [1.34–1.69]	< 0.0001
LDL-C, mmol/L, median [IQR]	1.43 [1.28–2.20]	1.55 [1.13–2.0]	0.96 [0.90–2.19]	3.20 [2.15–3.65]	0.0081
Apo-AI, mg/dL, median [IQR]	48.3 [18.8–53.2]	45.1 [18.6–56.9]	56.9 [48.2–61.6]	92.8 [84.9–103.9]	< 0.0001

Mann–Whitney test was performed to compare the lipid profiles of controls (n = 16) versus COVID-19 at admission (n = 8) patients.

TC, total cholesterol; TG, triglyceride; HDL-C, High-density lipoprotein cholesterol; LDL-C, Low-density lipoprotein cholesterol.

### Shotgun proteomics comparing HDLs from COVID-19 versus controls

**Figure 1:**
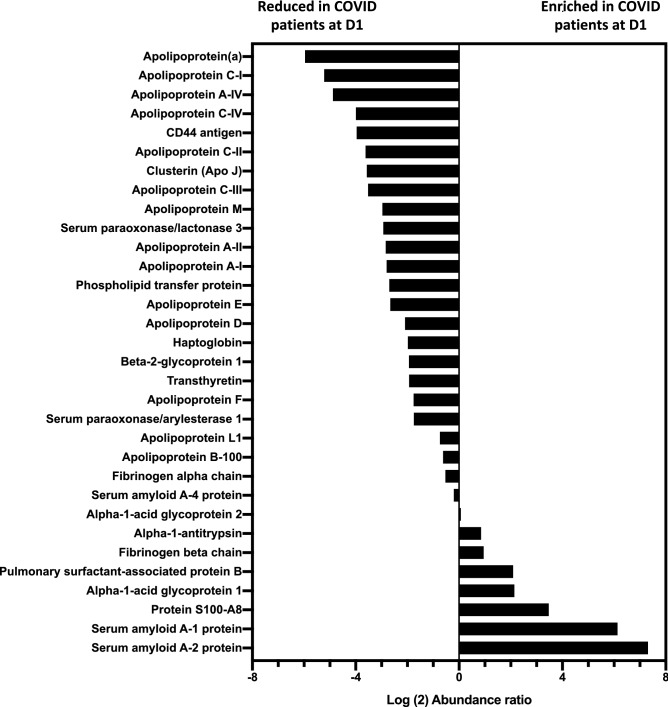
Relative abundance of proteins identified in HDLs isolated from of the plasma of control subjects (n = 15) and COVID-19 patients (n = 8) at admission in ICU (D1). The relative abundance of proteins in HDLs from controls and D1 COVID-19 patients was expressed as the Log2 Abundance ratio (D1 COVID/Control), as described in the “Methods” section.

**Figure 2:**
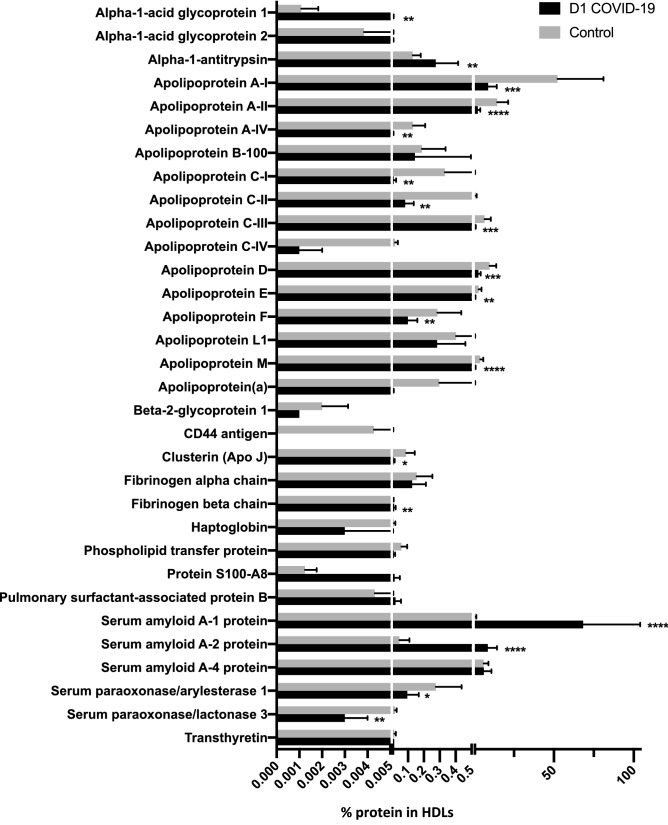
Percentage of protein abundance in HDLs from controls and COVID-19 patients. HDLs were isolated from the plasma of 15 controls and 8 patients with COVID-19 at admission (D1). Bar graphs represent the percentage of intensity ± SD of the two groups. Unpaired t-test was used. *p < 0.0332, **p < 0.0021, ***p < 0.0002 and ****p < 0.0001 as compared to the control group.

Trypsin digestion was performed on 3 μg total proteins and the resulting peptides were desalted and subsequently analyzed by LC-MS/MS as described in the “Methods” section. The following criteria were used in order to identify HDL-associated proteins in all samples (D1 COVID-19 and control HDL samples): *q-*values ≤ 0.01, unique peptides ≥ 2 and Xcorr ≥ 2. MS/MS spectra were searched against Uniprot Human database, using the Sequest HT search engine. For proteins with unique peptide below two, MS/MS spectra were manually inspected. Detailed information is given in the Supplementary Table 1. A total of 35 samples were analyzed from 8 COVID-19 patients at Day 1 and D3, 4 samples at D7 (n = 20 for COVID-19) and 15 controls. Shotgun proteomics allowed automated identification of 83 proteins in D1 COVID-19 + and control HDL samples (Supplementary Table 1). Among them, 32 proteins met the above-mentioned criteria allowing a high degree of confidence for identification (Table 3). All these proteins have already been previously found associated with HDL fractions using proteomic approaches^17,18^. We identified 5 proteins that were significantly more abundant in HDLs from COVID-19 patients, namely serum amyloid A-1 and 2 (SAA-1, 2), alpha-1 anti-trypsin (AAT), fibrinogen beta chain and alpha-1 acid glycoprotein. Conversely, 19 proteins were less abundant in COVID-19 versus control HDLs. In addition to ApoA-I, most of the apolipoproteins classically associated with HDLs were less abundant in COVID-19 HDL particles, including ApoA-II, ApoC-I, II, III, IV, ApoA-IV, ApoC-I, ApoJ, Apo(a), ApoE, ApoM, ApoD, ApoB100 and ApoF. Other proteins were also less abundant in HDLs from COVID-19 patients such as paraoxonases 1 and 3 (PON-1 and 3) (Table 3, Figs. 1 and 2). None of the proteins identified in HDLs were differential between D1 and D7 nor correlated with subsequent mortality (4 survivors vs 4 non-survivors, Supplementary Table 2). However, 6 proteins were significantly correlated with SAPSII, 5 of which were negatively correlated with severity (phospholipid transfer protein (PLTP), ApoAI, ApoAII, Serum Paraoxonase 3 and serum amyloid A1), whereas apo(a) was positively correlated with severity (Supplementary Figure 2).

**Table 3:** List of proteins identified in D1 COVID-19+ and control HDL samples.

Accession	Protein names	Coverage [%]	# PSMs^a^	Unique Peptides^b^	% patients with protein identified (D1)^c^	Q Peptides^d^
P02763	Alpha-1-acid glycoprotein 1	9	6	1	100	1
P19652	Alpha-1-acid glycoprotein 2	17	8	2	87.5	3
P01009	Alpha-1-antitrypsin	49	76	16	100	16
P02647	Apolipoprotein A-I	93	2167	54	100	54
P02652	Apolipoprotein A-II	77	348	15	100	15
P06727	Apolipoprotein A-IV	32	76	14	62.5	14
P04114	Apolipoprotein B-100	12	123	44	100	44
P02654	Apolipoprotein C-I	29	47	5	87.5	5
P02655	Apolipoprotein C-II	59	93	8	100	8
P02656	Apolipoprotein C-III	43	201	6	100	6
P55056	Apolipoprotein C-IV	29	10	3	37.5	3
P05090	Apolipoprotein D	42	649	14	100	14
P02649	Apolipoprotein E	78	335	28	100	28
Q13790	Apolipoprotein F	8	21	2	87.5	2
O14791	Apolipoprotein L1	34	108	13	87.5	13
O95445	Apolipoprotein M	53	203	14	100	14
P08519	Apolipoprotein(a)	47	82	29	87.5	29
P02749	Beta-2-glycoprotein 1	4	3	1	25.00	1
P16070	CD44 antigen	3	3	2	37.5	2
P10909	Clusterin (Apo J)	24	36	9	50.00	9
P02671	Fibrinogen alpha chain	20	94	12	100	12
P02675	Fibrinogen beta chain	4	14	3	87.5	3
P00738	Haptoglobin	21	22	2	62.5	2
P55058	Phospholipid transfer protein	11	30	4	75	4
P05109	Protein S100-A8	13	2	2	62.5	2
P07988	Pulmonary surfactant-associated protein B	5	5	2	50	2
P0DJI8	Serum amyloid A-1 protein	75	1114	8	100	14
P0DJI9	Serum amyloid A-2 protein	75	433	7	100	7
P35542	Serum amyloid A-4 protein	62	667	10	100	10
P27169	Serum paraoxonase/arylesterase 1	52	107	9	87.5	10
Q15166	Serum paraoxonase/lactonase 3	21	14	4	62.5	4
P02766	Transthyretin	33	9	3	37.5	3

Accession	Protein Abundance^e^ (PA) (Control)	Protein Abundance^e^ (PA) (D1 COVID)	Abundance ratio^f^ D1 COVID/Control	Log (2) Abundance ratio (D1 COVID/Control)	*p* values^g^	Variation (D1COVID vs Control)
P02763	1.28E + 04 ± 8.92E + 03	5.65E + 04 ± 4.08E + 04	4.41	2.14	0.0266*	↑
P19652	4.56E + 04 ± 5.79E + 04	4.80E + 04 ± 4.43E + 04	1.05	0.07	0.6355^ns^	–
P01009	1.55E + 06 ± 5.98E + 05	2.78E + 06 ± 1.43E + 06	1.80	0.85	0.0473*	↑
P02647	6.20E + 08 ± 3.42E + 08	8.91E + 07 ± 5.49E + 07	0.14	− 2.80	0.0003***	↓
P02652	1.72E + 08 ± 8.17E + 07	2.40E + 07 ± 1.55E + 07	0.14	− 2.84	0.0003***	↓
P06727	1.55E + 06 ± 9.17E + 05	5.28E + 04 ± 3.87E + 04	0.03	− 4.88	0.0025**	↓
P04114	2.22E + 06 ± 1.77E + 06	1.45E + 06 ± 3.57E + 06	0.65	− 0.62	0.0235*	↓
P02654	3.94E + 06 ± 3.29E + 06	1.06E + 05 ± 1.56E + 05	0.03	− 5.22	< 0.0001****	↓
P02655	1.02E + 07 ± 8.85E + 06	8.27E + 05 ± 5.47E + 05	0.08	− 3.62	0.0004***	↓
P02656	7.83E + 07 ± 4.66E + 07	6.82E + 06 ± 4.59E + 06	0.09	− 3.52	0.0002***	↓
P55056	2.30E + 05 ± 1.96E + 05	1.45E + 04 ± 6.42E + 03	0.06	− 3.99	0.0055**	↓
P05090	1.15E + 08 ± 5.03E + 07	2.71E + 07 ± 1.47E + 07	0.24	− 2.09	0.0003***	↓
P02649	3.44E + 07 ± 2.09E + 07	5.44E + 06 ± 3.51E + 06	0.16	− 2.66	0.0002***	↓
Q13790	3.39E + 06 ± 1.78E + 06	9.98E + 05 ± 6.12E + 05	0.29	− 1.76	0.0004***	↓
O14791	4.78E + 06 ± 3.13E + 06	2.87E + 06 ± 1.80E + 06	0.60	− 0.74	0.0755^ ns^	-
O95445	4.61E + 07 ± 2.25E + 07	5.87E + 06 ± 4.64E + 06	0.13	− 2.97	< 0.0001****	↓
P08519	3.53E + 06 ± 4.89E + 06	5.65E + 04 ± 5.42E + 04	0.02	− 5.96	0.0262*	↓
P02749	2.38E + 04 ± 1.35E + 04	6.18E + 03 ± 2.95E + 03	0.26	− 1.94	0.0762^ns^	–
P16070	5.08E + 04 ± 4.90E + 04	3.27E + 03 ± 2.78E + 03	0.06	− 3.96	0.011*	↓
P10909	1.05E + 06 ± 6.40E + 05	8.89E + 04 ± 6.64E + 04	0.08	− 3.57	0.0062**	↓
P02671	1.83E + 06 ± 1.18E + 06	1.27E + 06 ± 8.80E + 05	0.69	− 0.53	0.2651^ns^	–
P02675	7.19E + 04 ± 4.66E + 04	1.39E + 05 ± 9.18E + 04	1.93	0.95	0.2382^ns^	–
P00738	1.30E + 05 ± 1.22E + 05	3.29E + 04 ± 3.32E + 04	0.25	− 1.98	0.1068^ns^	–
P55058	6.97E + 05 ± 4.31E + 05	1.07E + 05 ± 8.26E + 04	0.15	− 2.70	0.0016**	↓
P05109	1.48E + 04 ± 6.13E + 03	1.64E + 05 ± 3.32E + 05	11.08	3.47	0.6905^ns^	–
P07988	5.13E + 04 ± 4.88E + 04	2.18E + 05 ± 3.42E + 05	4.25	2.09	0.5459^ns^	–
P0DJI8	9.86E + 06 ± 7.51E + 06	6.92E + 08 ± 3.61E + 08	70.16	6.13	< 0.0001****	↑
P0DJI9	5.51E + 05 ± 7.45E + 05	8.73E + 07 ± 5.81E + 07	158.33	7.31	< 0.0001****	↑
P35542	7.17E + 07 ± 3.45E + 07	6.22E + 07 ± 4.80E + 07	0.87	− 0.20	0.3572 ns	–
P27169	3.27E + 06 ± 1.93E + 06	9.69E + 05 ± 7.33E + 05	0.30	− 1.75	0.001**	↓
Q15166	2.12E + 05 ± 1.27E + 05	2.78E + 04 ± 1.36E + 04	0.13	− 2.93	0.0002***	↓
P02766	1.74E + 05 ± 1.05E + 05	4.56E + 04 ± 2.99E + 04	0.26	− 1.93	0.0324*	↓

HDL were isolated from the plasma of 15 control and 8 D1 COVID-19 subjects by ultrancentrifugation. MS/MS spectra were searched against Uniprot Human database, using the Sequest HT search engine. All protein identification and quantification was performed with *q-*values ≤ 0.01, unique peptides ≥ 2 and a Xcorr ≥ 2. For protein with unique peptide below two, MS/MS spectrum was manually inspected.

^a^PSM’s is the total number of identified peptide spectra matched for protein.

^b^Unique peptide to a protein identified from all samples in the analysis.

^c^% of patients for whom this protein has been identified at Day 1.

^d^Q peptide is the number of peptide used for the quantification.

^e^Protein abundance is the mean of total peptide intensities for each group.

^f^Abundance ratio is the ratio of the D1 COVID and Control protein abundances.

^g^*p* values were obtained by Mann–Whitney test.

*p < 0.0332, **p < 0.0021, ***p < 0.0002 and ****p < 0.0001 as compared to the control group.

**Figure 3:**
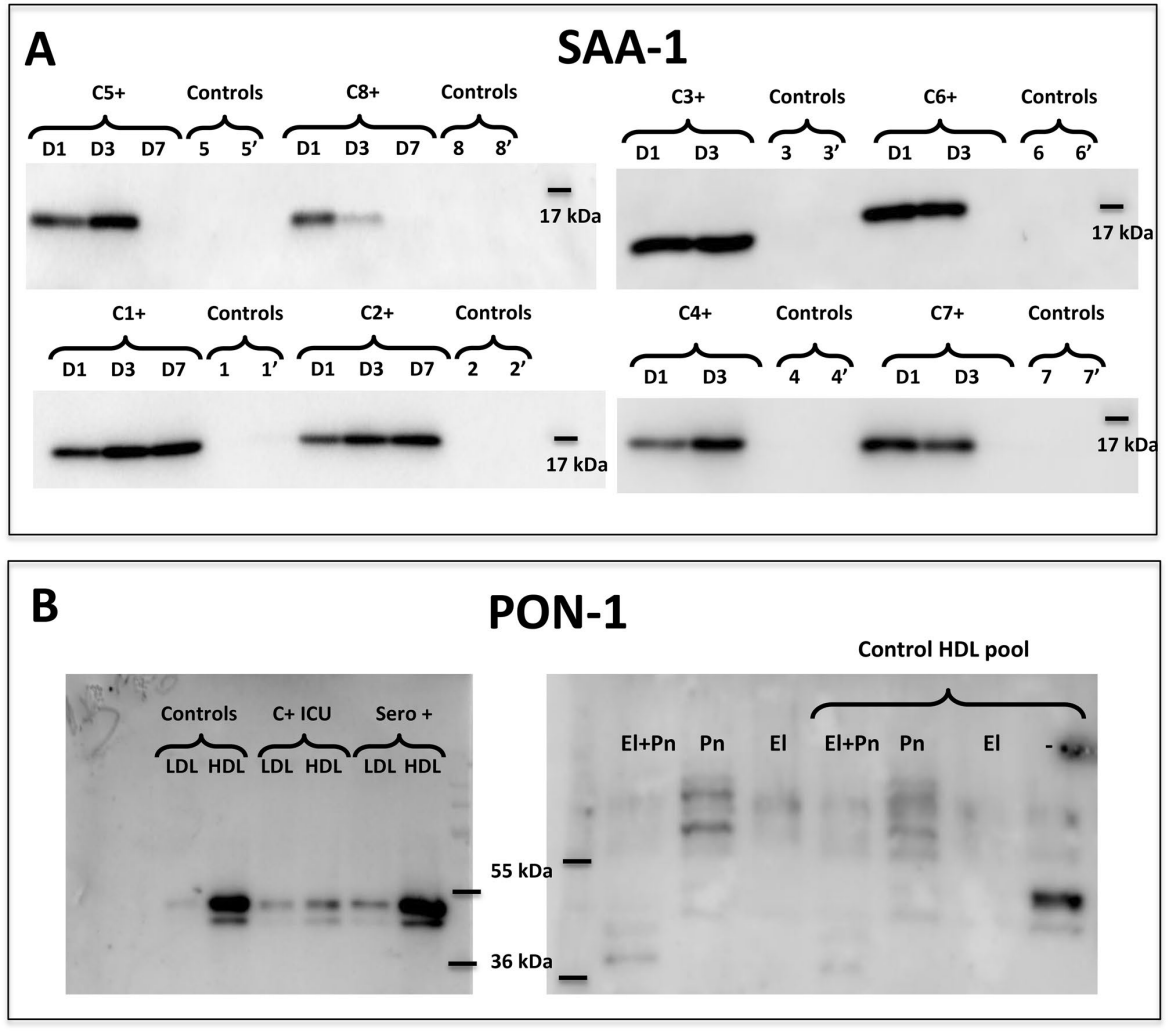
Western blot analysis for detection of SAA and PON-1 in HDL isolated from COVID-19 patients and controls. HDLs (3 μg protein /lane) were immunoblotted for SAA (serum amyloid A) (**A**) and PON-1 (paraoxonase 1) (**B**). (**A**) HDLs isolated from patients and controls are presented individually (C1+ = ICU-COVID-19 positive patient #1, C2+ for patient #2, etc.) at day 1, 3 and 7 after admission (D1, 3 and 7). Controls 1 and 1′ correspond to HDLs isolated from healthy subjects sex- and age-matched to patient #1. (**B**) Pools of LDLs and HDLs were blotted for PON-1 (left panel) after isolation from non-infected healthy subjects ("Controls"), COVID-19 positive patients in intensive care unit ("C+ICU") (n = 8) and SARS-CoV-2 seropositive cured caregivers ("sero+", sampled > 15 days after symptoms, n = 7). A pool of control HDLs containing PON-1 was incubated with elastase (El, 20 μg/mL), plasmin (Pn, 500 μg/mL) or both proteases (El + Pn) for 2 h at 37 °C. Enzymes alone (without HDLs) treated in the same experimental conditions were loaded as control. Uncropped gels are presented in Supplementary information file.

Western-blot analysis was performed on selected proteins in order to confirm mass spectrometry identification and abundance comparison between COVID-19 and control conditions: SAA-1, PON-1 (Fig. 3A,B) and AAT (Supplemental Figure 3). SAA-1 was abundantly associated with HDLs isolated from COVID-19 patients relative to that of controls. No trend was observed for SAA-1 abundance according to the number of days post-admission (D1, 3 and 7) and whether patients survived (C3+, C4+, C5+, C6 +) or not (C1+, C2+, C7+, C8+). PON-1 is known to be mainly transported by HDLs but can also bind to LDLs. We tested the presence of PON-1 by western-blot in LDL and HDL fractions from a pool of controls, severe COVID-19+ ICU patients (C+) and seropositive cured caregivers who did not need to be admitted in ICU (n = 4, Sero+). PON-1 is markedly less associated with HDLs from severe C+ ICU patients, thus confirming mass spectrometry results, whereas LDL-associated PON-1 was rather constant in all three groups (Fig. 3B). Since neutrophils are activated in COVID-19 and able to release proteases such as elastase, in association with a pro-thrombotic state involving the fibrinolytic system^19^, we tested the hypothesis that elastase and plasmin could degrade HDL-associated PON-1. Both proteases, alone or in combination were able to proteolyse PON-1 associated with HDLs from a pool of controls (n = 16) (Fig. 3B, right panel).

### Anti-inflammatory effects of HDLs on TNFα-stimulated human umbilical vein endothelial cells

**Figure 4:**
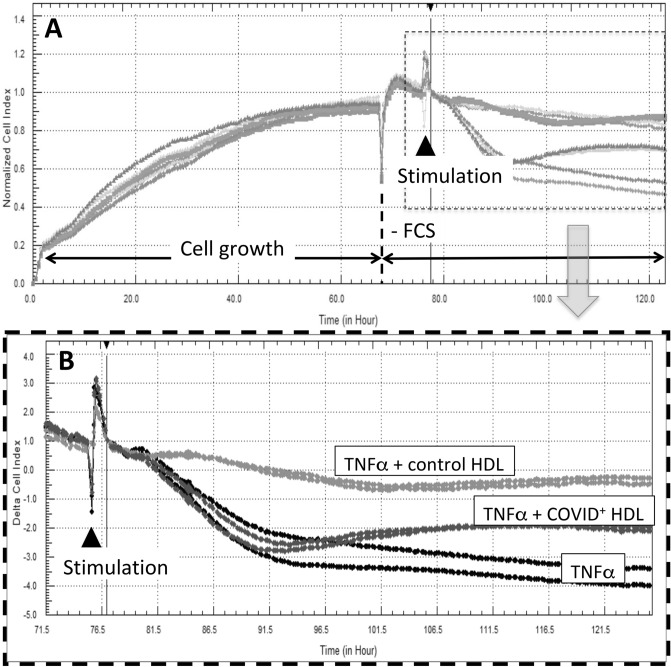
Real-time monitoring of HUVEC barrier dysfunction in response to TNFα+/−HDLs. Human umbilical vein endothelial cells (25,000 cells/well) were seeded on gelatin-coated xCELLigence 16 well E-plates. HDL were isolated from plasma of 16 controls and 8 D1 COVID ICU patients by ultracentrifugation and pooled into two groups: “Control HDL" and “COVID+ HDL”. The cell index was recorded continuously until confluence, when a plateau was reached [indicated cell growth in (**A**)]. Cells were then serum-deprived (- Fetal Calf Serum, FCS) for 6 h and then stimulated with 30 ng/mL TNFα+/−HDLs from COVID-19 patients or controls at 0.2 mg/mL. Cell indexes were normalized after stimulation [as indicated by the vertical line, in (**A**), 30 min. after the stimulation]. A magnification showing TNFα, TNFα + COVID + HDL and TNFα + control HDL is presented in (**B**). Representative results from 2 independent experiments. HDLs isolated from each patient and matched controls (2 controls/patients) were analyzed separately (Supplementary Fig. 4A).

Beyond their function as reverse transporter of cholesterol from peripheral tissues back to the liver, HDLs display endothelial protective properties^8^. We tested the anti-inflammatory effects of HDLs from patients or healthy subjects on HUVECs stimulated by TNFα. After 6 h serum deprivation allowing a better uptake of HDLs, HUVECs were incubated with 30 ng/mL of TNFα and the impedance was monitored for 36 h. Changes in cell index reflecting cell retraction and loss in cell–cell junctions was plotted over time as shown in Fig. 4. TNFα-induced decrease in cell index was prevented by co-incubation with HDLs from healthy subjects whereas HDLs isolated from COVID-19 patients displayed a reduced protective effect (% of HDL protection relative to TNFα expressed as median [Q1;Q3]: 24.50 [18.03;32.34] versus 17.07 [14.98;27.97] respectively in controls and COVID-19 patients, *p* = 0.0234, supplementary Fig. 4A). Figure 4 shows representative cell index curves of endothelial cells stimulated by TNFα alone and in the presence of pooled HDLs (n = 16 controls and n = 8 COVID-19). In the COVID-19 group, a trend towards a better protection of HDLs from survivors relative to non-survivor patients was observed (*p* = 0.057, Supplemental Figure 4B).

### Blunted anti-apoptotic effects of HDLs from COVID-19 patients in HUVECs stimulated by TNFα

**Figure 5:**
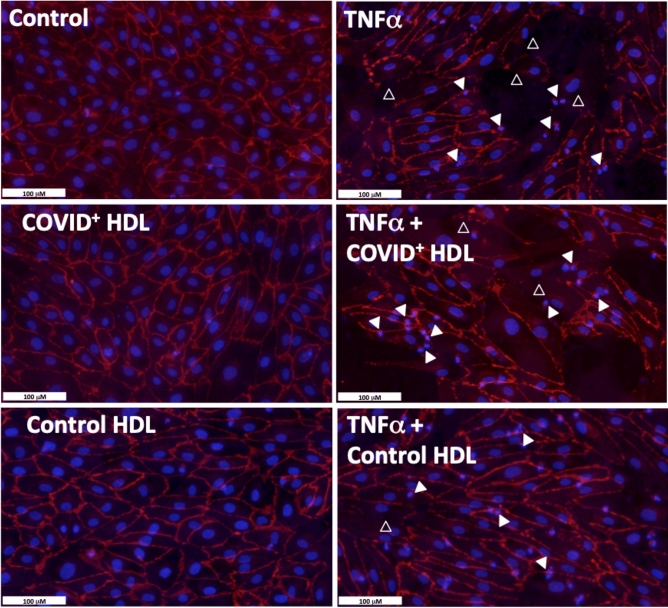
Effect of HDLs from controls and COVID-19 (C+) subjects on HUVEC apoptosis cells after stimulation with 30 ng/mL TNFα for 24 h. HDL were isolated from plasma of 16 controls and 8 D1 COVID-19 subjects by ultracentrifugation and pooled into two groups respectively “Control HDL" and “COVID+ HDL” Upper panel: immunostaining for VE-Cadherin (red) and nuclear staining with DAPI (blue). Empty white arrowheads show VE-Cadherin re-organization. Plain white arrowheads show examples of apoptotic nuclei (condensed and/or fragmented).

**Figure 6:**
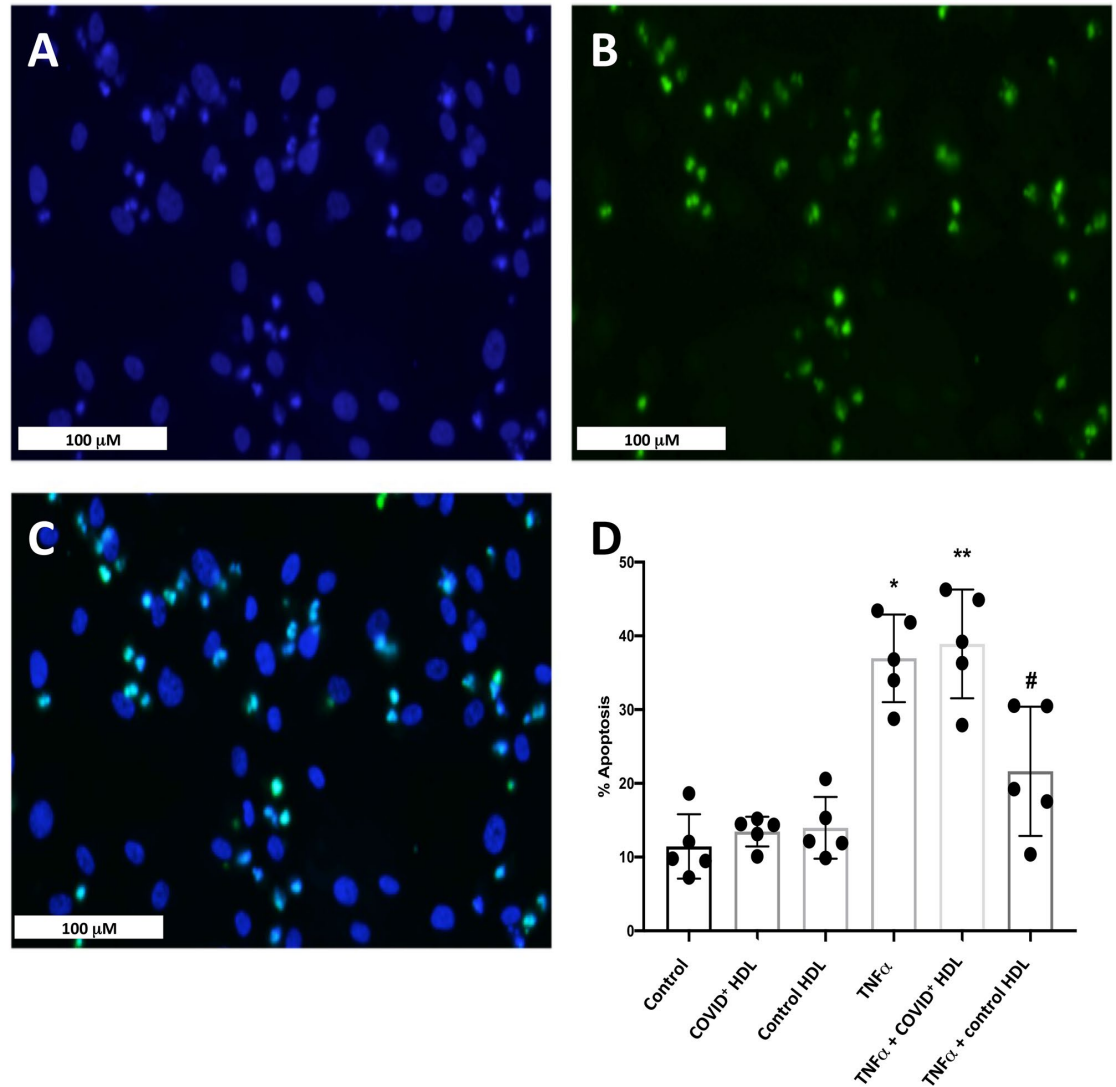
TUNEL and DAPI staining- Quantification of apoptotic nuclei. In TNFα-treated HUVECs (30 ng/mL 24 h), dual staining shows a good correlation between morphological apoptotic features determined by nuclear condensation and fragmentation (DAPI in blue, A, C) and DNA fragmentation (green, B, C). Bar graph (D) represents the means ± SD of apoptotic nuclei count in 5 fields (between 700 and 900 nuclei counted) of a representative experiment. *p = 0.0332 (Control vs TNFα). **p = 0.002 (Control vs TNFα + COVID+ HDL). *#p* = 0.03 (TNFα + control HDL vs TNFα + COVID+ HDL).

In addition, to prevent endothelial barrier permeability, HDLs are able to counteract apoptosis induced by TNFα^20^. After 36 h stimulation with TNFα, 37% of endothelial cells displayed characteristic apoptotic nuclear condensation and/or fragmentation^21^, as shown in Fig. 5 (plain white arrowheads) and Fig. 6 by TUNEL. A good correspondence was observed between nuclear changes quantified after DAPI staining and TUNEL (Fig. 6). HDLs from healthy subjects prevented apoptosis by 40% (reducing the % of apoptotic cells to 22%), whereas HDLs from patients were not able to thwart TNFα-induced apoptosis. In controls, the number of apoptotic nuclei corresponds to 11%, probably due to serum deprivation. Given that VE cadherin plays a key role in endothelial cell survival^22^, VE-cadherin immunostaining was performed (Fig. 5) and showed that TNFα induced a significant disorganization of cell–cell junctions characterized by a redistribution of VE-cadherin into the cytoplasm (empty white arrowheads). This disruption of cell–cell interactions by TNFα was, at least partially, prevented by co-incubation with HDLs isolated from non-COVID-19 subjects (HDL-) whereas HDL from COVID-19+ patients (HDL+) had no protective effect.

## Discussion

COVID-19 has emerged as a real threat to public health, characterized by damage to the respiratory tract, followed by an uncontrolled inflammatory response that can lead to organ failure and death^23^. A major impact of COVID-19 on the main metabolic parameters, and in particular on the lipid profile, has been reported by several authors, resulting mainly in a very significant drop in total cholesterol, LDL-C and HDL-C levels^2,4,6,24^. In the present study, we also observed very low HDL-C levels in patients as compared to controls and to normal values, whereas triglyceride concentration was doubled in COVID-19 patients. The prolonged use of Propofol (> 3 days) is reported to increase triglyceride levels in ICU patients^25^ and thus should not be the cause of the elevated levels observed at D1 in our study. Decreased levels of total cholesterol and cholesterol-transporting lipoproteins have been reported for a long time in ICU patients, particularly in bacterial sepsis, in which HDL particles may participate in lipopolysaccharide clearance^7,26^. In viral sepsis, the situation is less clear-cut even if lipid profile disorders have been reported in Dengue, HIV or Hepatitis infections^27–29^. A recent study in COVID-19 patients reports reduced plasma ApoA-I, ApoA-II and Apo-B levels as well as decreased LDL particle number and HDL subfractions assessed by nuclear magnetic resonance^30^. They also report increased LDL triglycerides and of VLDL parameters (particle number, free cholesterol and triglycerides) in these patients. Decreased HDL-C was also recently reported to be associated with the severity of COVID-19^4,31^.

Beyond quantitative aspects reporting levels of LDL-C, HDL-C and total cholesterol, very few studies focus on lipoprotein composition and functionality. High-density lipoproteins are important for cholesterol metabolism, but also display major pleiotropic functions that could play a pivotal role in acute inflammatory conditions. A large body of evidence suggests that HDLs are globally protective for the endothelial layer, due to their antioxidant, anti-inflammatory, anti-apoptotic and anti-thrombotic functions^8^. HDL endothelial protective effects have been shown to be crucial in bacterial sepsis or lung emphysema, in which supplementation with functional HDLs or ApoA-I particles limited the deleterious effects of acute inflammation^32,33^. Both HDL composition and functionality are profoundly modified under pathological conditions, in cardiovascular disease^11,13^ and in endotoxemia inflammation^34^. A recent proteomic analysis of plasma from septic patients secondary to community-acquired pneumonia and hospital acquired pneumonia demonstrated that proteins associated with HDLs were markedly decreased, including ApoA-I, C-I, A-IV, L1 and paraoxonase 1 whereas proteins of the acute phase were increased (SAA, CRP, etc.) relative to control subjects^35^. In the present study, we aimed at characterizing HDL protein composition and endothelial protective effect by a mass-spectrometry-based proteomic approach in COVID-19 patients. HDL fraction was isolated by ultracentrifugation, starting form a small volume of plasma (400 μL). Although this method is the gold standard for lipoprotein isolation, some proteins may be lost during the two steps of ultracentrifugation, and the sensitivity for proteomic identification may be insufficient to discover low-abundance proteins, due to the small amount of plasma initially available. However, under these conditions, we identified 83 proteins associated with HDLs. Among these proteins, most of the expected apolipoproteins associated with HDL were decreased, including ApoA-II, ApoC-I, II, III, IV, ApoA-IV, ApoC-I, ApoJ, Apo(a), ApoE, ApoM, ApoD, ApoB100 and ApoF. In addition, other proteins including beta2-glycoprotein 1, CD44 antigen and serum paraoxonases 1 and 3 were also less abundant in HDLs from COVID-19 patients. Paraoxonase 1 plays an important role in antioxidant HDL functions and was shown to be decreased in septic patients^36,37^. Here, we show that PON-1 is less abundant on HDL particles isolated from COVID-19 patients. We tested the possibility of a proteolytic degradation of this antioxidant enzyme by elastase and plasmin. Neutrophil activation during COVID-19 produces elastase release potentially associated with neutrophil extracellular traps as well as an increased thrombus formation, leading to production of plasmin^19,38^. Both enzymes incubated with HDLs isolated from healthy subjects were able to degrade PON-1, suggesting that plasmin and/or elastase may participate in the decreased levels of HDL-associated PON-1. Conversely, other proteins were found to be more abundant in HDLs from COVID-19 patients, such as SAA-1 and 2, alpha-1 antitrypsin (AAT) and alpha-1 acid glycoprotein 1, considered as acute phase proteins. In a previous study, we reported an increased abundance of AAT associated with HDLs from ischemic stroke patients^11^. Here we have confirmed the proteomic results by western blot (Supplemental Figure 2) for AAT and SAA-1 that were clearly markedly associated with HDLs from COVID-19 patients. SAA has been shown to represent the most abundant protein of HDLs at the start of the sepsis and is then slowly replaced by ApoA-I during recovery^39^. In COVID-19, plasma SAA has recently been suggested as a biomarker of severity; patients with high initial levels of SAA levels were more likely to have poor computed tomography imaging^40^. SAA was suggested as a predictor of prognosis in patients with COVID-19, being significantly higher in non-survivors versus survivors, with a higher sensitivity relative to CRP^41^. In the present study, no difference was observed between survivors and non-survivors for any protein identified in HDLs from COVID-19 patients; this may be due to the small number of patients (4 survivors and 4 non-survivors).

Surfactant-associated protein B (SAPB) was found in HDLs but was not significantly more abundant in COVID-19 patients, albeit a trend was observed. SAPB has been identified previously in HDLs and shown to impair their antioxidant capacity^42^. SABP has also been identified in HDLs from patients with end-stage renal disease, along with SAA and Apo C-II; these particles had reduced anti-inflammatory capacity^43^. High levels of HDL-associated SAPB were also found to predict mortality in heart failure patients^44^.

ApoA-I levels are significantly decreased in COVID-19 patients, suggesting a decreased synthesis by the liver and/or its replacement by SAA in HDLs. A recent study also reports decreased serum HDL-associated apolipoproteins in COVID-19 patients including Apo-AI, Apo-AII, Apo-H, Apo-L1, Apo-D and Apo-M; they also show an important increase in SAA-1 and 2^45^. In inflammatory conditions, SAA synthesis by the liver is increased and this acute phase protein was shown to associate with dense HDL particles (HDL_3_)^46,47^. We then tested potential correlations between HDL-associated protein abundance and the severity of COVID-19, assessed using SOFA (Sepsis-related Organ Failure Assessment) score or SAPSII (Simplified Acute Physiology Score II). Negative correlations were observed between severity at admission (SAPSII) and ApoA-I, ApoA-II, phospholipid transfer protein (PLTP), serum paraoxonase 3 and SAA1, whereas apolipoprotein(a) was positively correlated with SAPSII. Apo(a) has been suggested to be an aggravating factor in COVID-19 due to its pro-thrombotic properties^48^, but there is currently no experimental or clinical data supporting this hypothesis. No correlation was observed between the SOFA score and any of the proteins identified in HDLs. This score is mainly related to sepsis and may not be very discriminating between patients, since COVID-19 is initially characterized by a severe pneumonia.

In addition to important modifications in protein cargo, we demonstrate that HDLs from COVID-19 patients were less protective for endothelial cells stimulated with TNFα than HDLs from healthy subjects. During COVID-19, increased endothelial permeability is expected to play an important role in organ failure subsequently to the SARS-Cov2 cytokine inflammatory storm^49^. We tested the protective effects of HDLs from patients and controls in HUVECs stimulated with TNFα. Endothelial barrier dysfunction was monitored by xCELLigence real-time cell analysis. Endothelial adherent cells impede the electron flow (impedance); this parameter is expressed as arbitrary units called cell index, and shown to decrease in case of detachment and increased permeability^50^. HDLs from COVID-19 patients displays a blunted protective effects against HUVEC permeability, VE-cadherin peripheral belt the cell–cell disruption and apoptosis induced by TNFα. Interestingly, HDLs isolated from COVID-19 survivor patients showed a trend towards a better protection than HDLs from non-survivors (*p* = 0.057).

One limitation of our study could be the low number of COVID-19 patients tested. HDLs were isolated from each patient and tested individually or as a pool for their anti-inflammatory capacity and by western blot. These techniques are suitable only for a limited number of samples. COVID-19 and controls were well matched for sex and age and two controls were included for each case. The magnitude of the difference observed between groups is compatible with the small number of samples tested (in total 20 COVID-19 and 16 controls). Due to the limited amount of HDLs, we did not test for the reverse cholesterol transport capacity of HDLs from COVID-19 *versus* healthy subjects, but rather their anti-inflammatory and anti-apoptotic functions on endothelial cells. These properties are of particular importance in the context of COVID-19 in which the endothelium is exposed to the inflammatory cytokine storm^9^.

The main findings of our study are that HDL particles from patients suffering COVID-19 are characterized by quantitative and qualitative abnormalities relative to those isolated from healthy subjects. Low levels of HDLs in ICU severe COVID-19 patients associated with an important dysfunction, characterized by a loss of protective effect towards endothelial cells in inflammatory conditions, supports the relevance of a potential therapy relying on HDL supplementation during the acute phase of COVID-19.

## Methods

### Study population

COVID-19 patients were enrolled in a monocentric study conducted in the surgical ICU of Bichat Claude-Bernard University Hospital, Paris, France. Patients admitted for sepsis due to COVID-19 pneumonia were consecutively and prospectively included. The study was approved by our local ethics committee (Comité de Protection des Personnes Ile-de-France n° 1, Aposize study, RCB: A02267-46). Patient demographics, Simplified Acute Physiology Score II (SAPSII), Sepsis-related Organ Failure Assessment (SOFA) severity scores and clinical data were collected. Mortality at 28 days, duration of mechanical ventilation, number of days alive without mechanical ventilation at day 28, length of stay in the ICU and in the hospital, renal replacement therapy, vasopressor use, need for extracorporeal membrane oxygenation (ECMO), and tracheostomy were collected. A number of prone positioning procedures and ventilator-associated pneumonia were also collected. At admission (D1), D3 and D7, 5 mL EDTA blood samples were collected and the plasma was stored at − 80 °C. Plasma concentrations of total cholesterol (TC), HDL-C, LDL-C, and triglycerides (TG) were also measured at each time (D1, D3 and D7) in the Biochemistry Laboratory of Bichat Claude-Bernard Hospital by routine enzymatic assays (CHOL, HDL-C, LDL-C and TRIG methods, Dimension VISTA System, Siemens Healthineers). The reference values for these assays were as follows: HDL-C: > 1.40 mmol/L; TC: 4.40 < N < 5.2 mmol/L; and triglycerides: 0.50 < N < 1.7 mmol/L. According to the recommendations of the French National Authority for Health 2017 and the European Society of Cardiology 2016, LDL-C concentration targets have been established depending on vascular risk factors^51^. Control plasma from healthy volunteers were sampled from caregivers of the University Hospital Center of Reunion Island, after obtaining ethical committee approval (Comités de Protection des Personnes, Nord Ouest IV de Lille, France; number EudraCT / ID-RCB 2020-A01253-36). All participants gave their informed consent and were fasting before venous puncture. Blood was sampled in 10 mL EDTA tubes and plasma was stored at − 80 °C. Age, gender, body mass index (BMI) and comorbidities were collected for all participants. All methods were carried out in accordance with relevant guidelines and regulations.

### Lipoprotein isolation from plasma

Lipoproteins were isolated from EDTA-plasma by a classical 2-steps sequential density ultracentrifugation as described previously^52^. All the following procedure has been performed under a Class II microbiological safety workbench. Briefly, the density of plasma was adjusted to 1.063 using potassium bromide (KBr): 99 μL of KBr solution (d = 1.35) were added to 500 μL of plasma, which was then underlaid to 1 mL of KBr saline solution (d = 1.063). The 2 mL tube was then filled with 300 μL of KBr solution (d = 1.063). Ultracentrifugation was performed at 250,000 g for 20 h at 10 °C in a 50.4Ti rotor using a Beckman Coulter Optima L-80 XP Ultracentrifuge. The upper lipoprotein fraction containing LDLs (orange layer) was recovered as a single band, and the KBr was eliminated by 3 washing steps (3 × 500 μL of Saline EN (0.9% NaCl, 1 mM EDTA, and 0.025% NaN_3_)) using a centrifugal filter device (Amicon Ultra, 3 KDa cut off, UFC500396). The density of the bottom fraction (150 μL) resulting from the first ultracentrifugation and containing HDLs was adjusted to 1.21 g/mL with KBr (by adding 525 μL of KBr d = 1.35) and overlaid with 1 mL of KBr saline solution (d = 1.21). The second ultracentrifugation and subsequent washing steps were similar to those for the 1.063 lipoprotein fraction. 150 μL of HDL fraction were recovered, desalted and concentrated to 100 μL with saline EN solution. The total protein concentration was measured in triplicate using a Bradford Ultra kit (Expedeon, Cambridge, UK, EX-BFU05L). The purity of the HDL fraction was evaluated by sodium dodecyl sulfate- 12% polyacrylamide gel electrophoresis (SDS-PAGE) and western blot analysis using rabbit anti-ApoA-I polyclonal antibody (Abcam, ab52945 used at 1:4,000 = 47.5 ng/mL).

### HDL sample preparation for mass spectrometry

HDL total protein concentration was determined by using the BradfordUltra test, following the manufacturer's instructions. Bovine serum albumin was used as standard. Briefly, 3 µg of isolated HDL were diluted in 300 μL of digestion buffer (50 mM ammonium bicarbonate) and then reduced with 20 mM DTT for 20 min at 60 °C before alkylation with 40 mM iodoacetamide (30 min at room temperature in the dark). Samples were subjected to proteolytic digestion with 3 µg of trypsin-tosylphenylalanyl chloromethyl ketone (Sigma) overnight at 37 °C. Digested peptides were acidified with 20% trifluoroacetic acid. After 15 min at 4 °C, peptides were collected by centrifugation at 10,000×*g* for 15 min at 10 °C and desalted using Pierce peptide desalting spin columns (Thermo Fisher Scientific, part no. 2162704). Eluted peptides were dried in a Speed Vacuum before being concentrated and purified again using C18 Tips (Thermo Fischer Scientific, part no. 87782). Peptides were eluted with 20 µL of 40% acetonitrile in 0.1% TFA and dried under vacuum prior to LC-MS/MS analysis.

### NanoLC–MS/MS analysis

The tryptic peptide digests were resuspended in 7 µL of 4% acetonitrile (ACN) in 0.1% TFA and analyzed by nano-LC using a Thermo Fisher Ultimate 3000 series NCS-3500 RS coupled with NSI-Q-Orbitrap mass spectrometer (Q Exactive Plus, Thermo Fisher Scientific, Bremen, Germany). Briefly, 5 µL of sample were separated on a LC-EASY-spray C18 column (2.6 µm, 100 Å, 75 µm × 25 cm, Thermo Fisher Scientific). Peptides were eluted using two steps linear gradient from 4 to 27.5% solvent B (0.1% formic acid in 80% ACN) for 127 min and then from 27.5 to 44% solvent B for 18 min. A washing step was then carried out with 90% solvent B for 5 min followed by an equilibration step with 4% solvent B for 25 min. Solvent A was 0.1% formic acid in water. The column temperature was held at 40 °C and the flow-rate was set to 300 nL/min. The mass spectrometry analysis was performed with the following conditions: spray voltage 2 kV, heated capillary temperature: 275 °C and S-lens RF level: 30%. Mass spectra were acquired with XCalibur 4.2.47 software (Thermo Fisher Scientific) and registered in data-dependent acquisition with the mass spectrometer operating in positive mode. Survey full scan mass spectra were acquired in the 350 to 2,000 m*/z* range at a resolving power of 70,000 (at m/z 400) with an automatic gain control (AGC) target of 1e^6^ and maximum injection time (max IT) of 120 ms. Top 10 precursors were selected for MS/MS spectra, with a resolution at 17,500 (at *m/z* 400), AGC target of 1e^5^ and max IT of 64 ms. Peptides were fragmented by higher energy collisional dissociation (HCD, 28% normalized collision energy). Dynamic exclusion was activated with a repeat duration of 60 s. Charge state screening was enabled, and precursors with either unknown or 1^+^ charge states were excluded. Injection of blank (4% acetonitrile in 0.1% TFA) was performed before and after samples to prevent carry-over.The Orbitrap performance was evaluated weekly and external calibration of the mass spectrometer was performed prior to analysis with a LTQ ESI positive ion calibration solution (Pierce).

### Protein identification and quantification

Raw mass spectrometry data were automatically processed using Proteome discoverer software (version 2.2.2.2.0, Thermo Fisher Scientific) for protein identification and quantification. MS and MS/MS spectra were searched against Uniprot human reference proteome database with canonical and isoform sequences (20,421 sequences; 04 June 2019) using SEQUEST HT search engine. The database search was performed with the following parameters: oxidized methionine and protein N-terminal acetylation were set as variable modifications, and Cysteine carbamidomethylation was set as a fixed modification. Trypsin was set as enzyme specific and two missed cleavages allowed. A mass tolerance of 10 ppm was used for precursor ions and 0.02 Da for product ions. The false-discovery rate (FDR) was fixed to 1% at the level of proteins and peptides using a target-reversed decoy database search strategy. A minimum of one unique peptide sequence with a Sequest score (Xcorr) ≥ 2 was used. For peptides with an Xcorr < 2, identification was confirmed by manual interpretation of corresponding MS/MS spectrum. A label-free quantification was performed on identified proteins by using the peak intensities of validated peptides for a given protein. Both unique and razor peptides were used for quantification. The list of proteins and peptides used for identification and quantification is shown in Supplementary material.

### Data processing

All computations were calculated with Excel (Microsoft) and Proteome discoverer software (version 2.2.2.2.0). Group protein abundance was defined as the mean of protein intensity of all samples for each group (D1 COVID+ vs control). Protein intensity was defined as the sum of peptide intensities of the considered protein for each individual. Abundance ratio is the ratio of the protein abundance of the COVID group to that of control subjects. Then, the Log (2) abundance ratio was calculated to show differences between groups.

### Western-blot analysis

Classical SDS-PAGE migration (12% polyacrylamide) was carried out in order to separate the proteins contained in the HDL fraction, followed by a liquid transfer to nitrocellulose membrane, as previously described^52^. The following primary antibodies were used: anti-paraoxonase 1 (Abcam, ab24261, used at 1:1000 dilution = 2 μg/mL), anti-serum amyloid A-1 (Abcam, ab190802, used at 1:10,000 dilution = 42 ng/mL), anti-alpha-1 antitrypsin (Calbiochem, catalog no. 178260, used at 1:1,000), anti-ApoA-I (Abcam, ab52945 used at 1:4,000 = 47.5 ng/mL).

### Real-time monitoring of HUVEC barrier dysfunction

Human umbilical vein endothelial cells (HUVECs) were obtained from human umbilical cords of patients who delivered babies at Sainte-Clotilde Clinic (Saint-Denis de La Réunion, France), with informed consent, according to French Law L.1243-3 modified by articles R1243-49 to 56, requiring the declaration of "Biobanking and preparation of cells and tissues from human body for research purpose" to MESR (French higher education and research ministry), Inserm (French National Institute for Health and Medical Research), and ANSM (French National Agency for Medicines and Health Products Safety) with the following references. Inserm: C 19-23, IDRCB: 2019-A01137-50, and MESR: DC-2016-2614. HUVECs were obtained from three human donors. Umbilical cords were resected rapidly after birth, and immediately stored in sterile saline solution (PBS). HUVECs were detached from umbilical veins using collagenase NB6 solution (Nordmark biochemical, N0003224, used at 5 mg/mL) for 10 min à 37 °C. Then, cells were cultured in complete endothelial cell growth medium EGM-2 (Lonza, Basel, Switzerland) at 37 °C in 5% CO_2_ and maintained using standard cell culture. After reaching confluence, HUVECs were detached using 0.25% trypsin-EDTA and seeded at a concentration of 25,000 cells/well on gelatin-coated xCELLigence 16 well E-plates (Acea Biosciences, San Diego, CA, United States). Cell index was recorded continuously during cell growth until confluence, when a plateau was reached (until 70 h). HUVECs were serum-deprived for 6 h before stimulation with TNFα (10 ng/mL) in the presence or not of 50 μg/mL HDLs from COVID-19 or control subjects. Cell index measurements were automatically collected every 15 min from 0 to 120 h.

### TUNEL reaction and VE-Cadherin immunostaining

HUVECs were seeded at 50,000 cells/well in Millicell 8-well EZ Slides (Merck, Molsheim, France) in complete EGM-2 medium supplemented with 5% fetal calf serum. After reaching confluence, HUVECs were serum-deprived for 6 h before stimulation with TNFα (10 ng/mL) in the presence or not of 50 μg/mL HDLs from COVID-19 or control subjects for 24 h. Cells were rinsed with PBS and then fixed with 3.7% paraformaldehyde for 15 min at room temperature. Terminal transferase dUTP nick-end labeling reaction was performed according to the manufacturer's instructions (Roche, Cat. No. 11 772 465 001). After 5 min wash with PBS, cells were permeabilized with 0.1% Triton X100 in 0.1% sodium citrate for 2 min on ice. Positive control was realized with 500 U/mL DNAse I for 10 min at 20 °C. After washing with PBS, the reaction mixture containing fluorescein-labelled dUTP was added to the wells for 2 h at 37 °C in a humidified chamber. For VE-cadherin immunostaining, non-specific antigenic sites were blocked by 2.5% BSA in PBS and the slides were then incubated with anti-VE-Cadherin antibody (Abcam, Ab33168) at 1 μg/mL in 1% BSA for 90 min at room temperature in a humidified chamber. After 3 washes with PBS, a goat anti-rabbit conjugated to Alexa 594 nm was applied for 1 h at RT, in PBS containing 1% BSA. The slides were rinsed 5 times with PBS, incubated with 0.1 μg/mL DAPI for 10 min at RT, washed with PBS and finally with water before mounting in fluorescence medium and image captured using a NanoZoomer S60 digital slide scanner.

### Statistical analysis

Statistical analysis was carried out with Prism (GraphPad Software Inc., San Diego, CA, USA). Continuous variables were expressed as medians with interquartile ranges (IQRs). Statistical significance was assessed using a Mann–Whitney test to compare COVID-19+ patients versus controls, unpaired t-test to compare percentages and Wilcoxon paired test when appropriate. *p* values < 0.05 were required for significance. Spearman test was used for non-parametric assessment of correlations.

## Supplementary Information

Supplementary Information 1.

Supplementary Information 2.

Supplementary Information 3.

Supplementary Information 4.
