# Supplementary material for: Altered high-density lipoprotein composition and functions during severe COVID-19
Source: Sci Rep. 2021 Jan 27;11:2291. doi: 10.1038/s41598-021-81638-1 (PMC7841145; doi:10.1038/s41598-021-81638-1)
Supplement: Supplementary file 1 — Supplementary Information 1. [file 41598_2021_81638_MOESM1_ESM.pdf]

Altered high-density lipoprotein composition and functions during severe COVID-19

Floran Begue[1], Sébastien Tanaka[1,2], Zarouki Mouktadi[1], Philippe Rondeau[1], Bryan Veeren[1], Nicolas Diotel[1], Alexy Tran-Dinh[2,3], Tiphaine Robert[4], Erick Vélia[5], Patrick Mavingui[6], Marie Lagrange-Xélot[7], Philippe Montravers[2,3,8], David Couret[1,9,+] and Olivier Meilhac[1,10,*,+]

[1] Université de La Réunion, INSERM, UMR 1188 Diabète athérothombose Réunion Océan Indien (DéTROI), Saint-Denis de La Réunion, France.

[2]AP-HP, Service d'Anesthésie-Réanimation, CHU Bichat-Claude Bernard, 75018 Paris, France.

[3]Université de Paris, UFR Denis Diderot, Paris

[4]AP-HP, Service de Biochimie, CHU Bichat-Claude Bernard, 75018 Paris, France.

[5] Clinique Sainte-Clotilde, Groupe Clinifutur, Pôle mère enfant, 97490 Sainte-Clotilde, La Réunion, France.

[6] Université de La Réunion, UMR Processus Infectieux en Milieu Insulaire Tropical (PIMIT), INSERM 1187, CNRS 9192, IRD 249, 2 rue Maxime Rivière (GIP CYROI), 97490 Sainte-Clotilde, La Réunion, France.

[7] CHU de La Réunion, Service des maladies infectieuses, Saint-Denis, France.

[8] Inserm UMR 1152 Physiopathologie et épidémiologie des maladies respiratoires, Université Diderot, Paris

[9] CHU de La Réunion, Service de neuro-réanimation, Saint-Pierre, France.

[10] CHU de La Réunion, CIC-EC 1410, Saint-Pierre, France.

* Corresponding author: olivier.meilhac@inserm.fr

[+]These authors contributed equally to this work

Running Title: HDL in COVID-19

Supplementary Figure 1

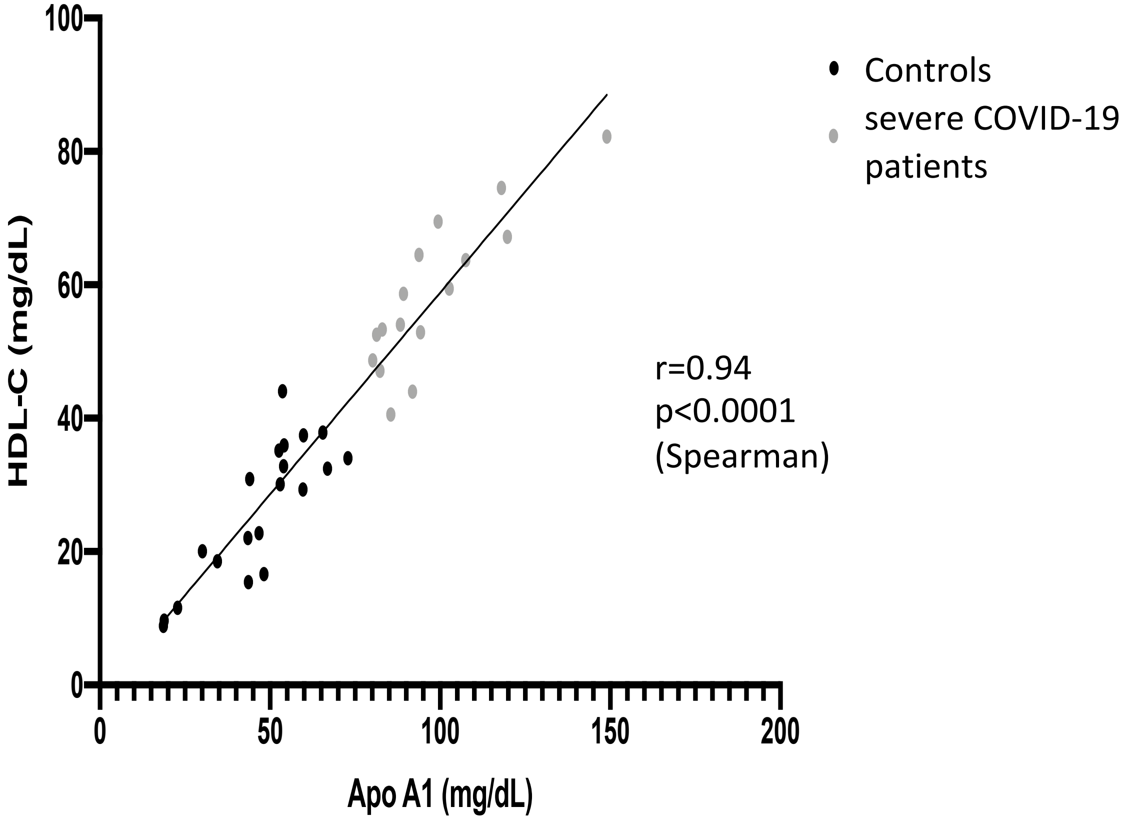

Correlations between HDL-cholesterol (HDL-C) levels and ApoA-1 concentration in plasma assessed by quantitative mass spectrometry. Controls are represented as grey circles and COVID-19 patients as black circles (all D1, D3 and D7 time points).

Supplementary Figure 2

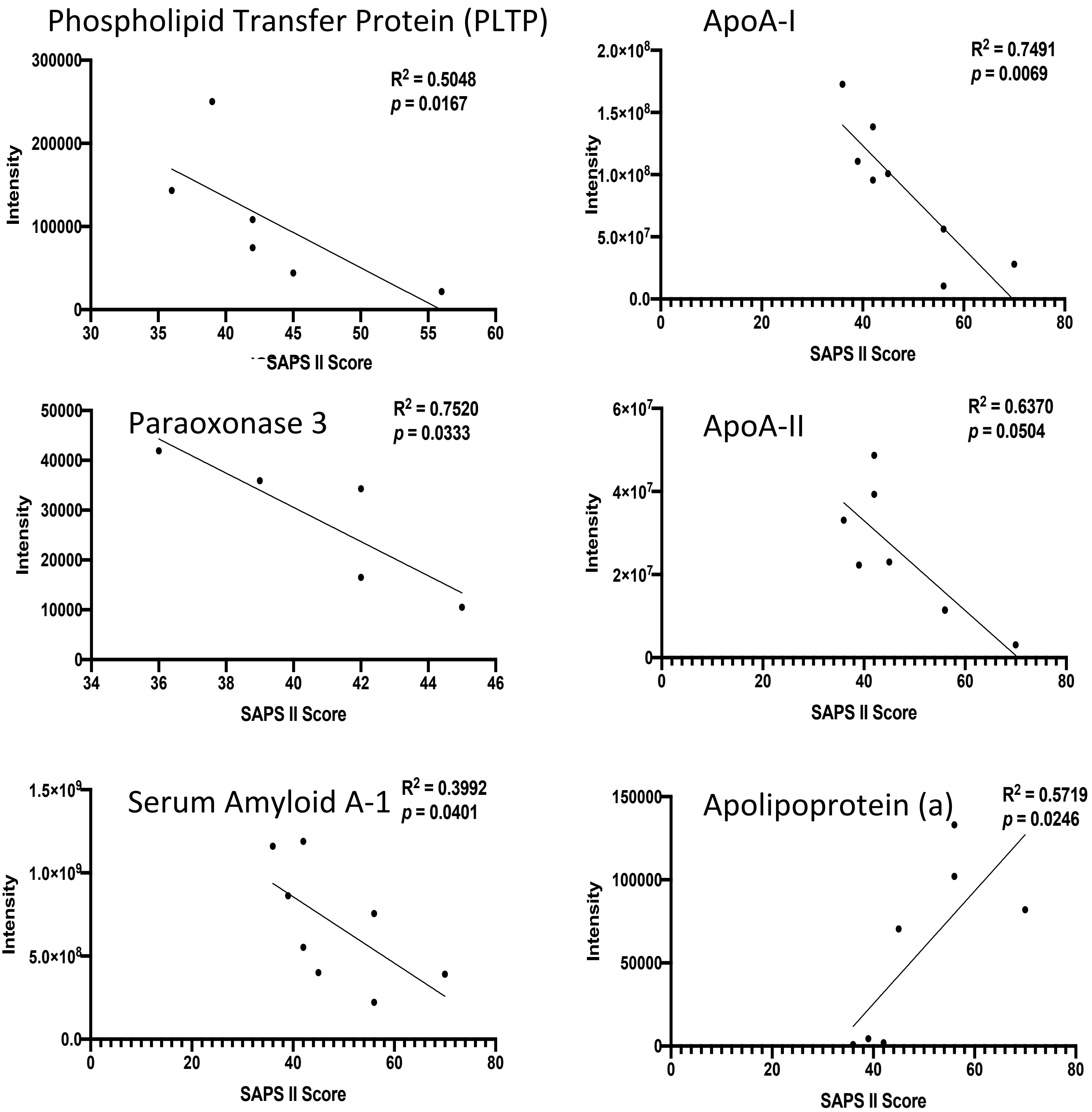

Protein abundance at day 1 as a function of SAPSII (Simplified Acute Physiology Score II). Protein intensity determined by MS analysis was plotted against SAPSII (severity score at admission). Spearman correlation test was performed and only proteins with p value ≤ 0.05 are presented.

Supplementary Figure 3

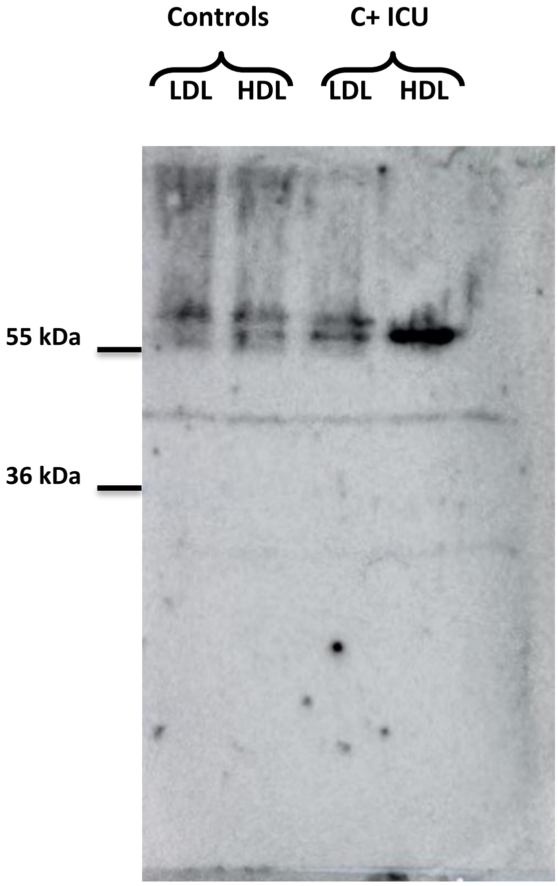

Western blot analysis against alpha1-antitrypsin of pooled LDLs and HDLs isolated from control subjects or severe COVID-19 patients: 3mg of total proteins were separated by SDS-PAGE, transferred and probed using anti-alpha1 antitrypsin antibody, followed by incubation with horse-radish peroxidase-conjugated secondary antibody and chemiluminescence reaction with ECL-Prime.

Supplementary Figure 4

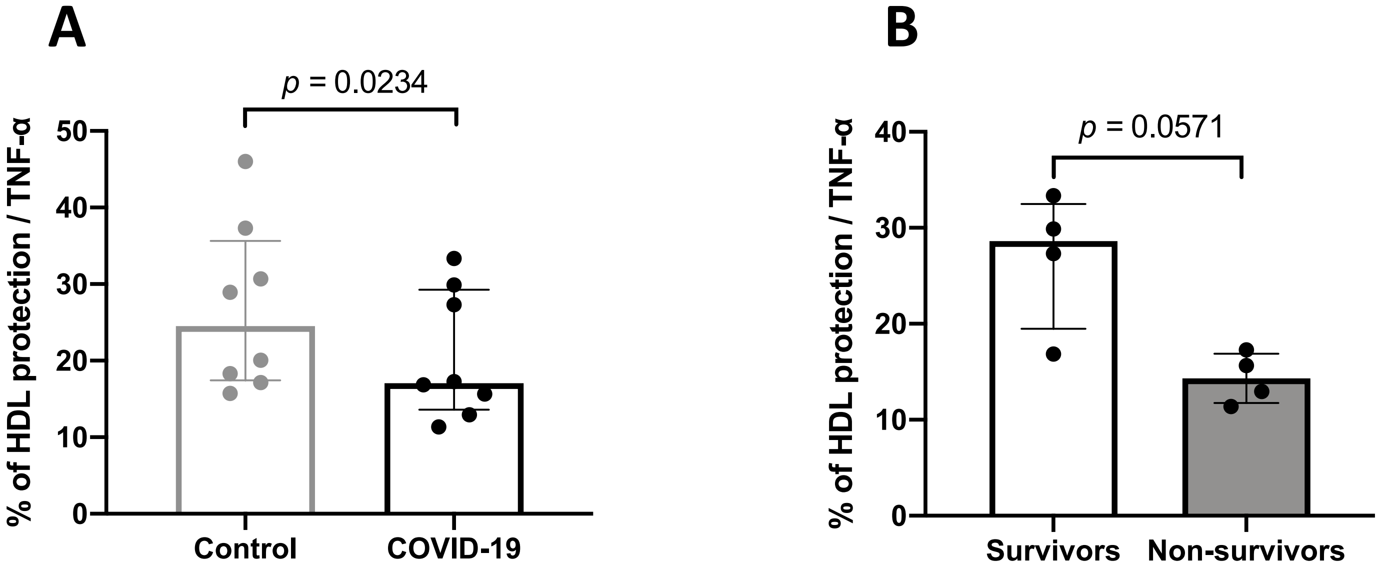

Protection of TNFα-induced changes in endothelial cell impedance by HDLs isolated from controls vs COVID-19 patients (A) and from survivors vs non-survivors COVID-19 patients (B). The percentage of protection was calculated as follows: [(AUC *100)/ Mean (AUC TNF-α)]–100, where AUC represents the area under the curve during the 20 hours following stimulation with TNF-α. (A) HDLs were isolated from each COVID-19 patient and from age- gender-matched controls (HDLs from 2 controls were pooled and compared with each corresponding patient). (B) HDLs from survivors COVID-19 patients were compared to HDLs from non-survivors (n=4/group). Results are expressed as median +/- IQR. p values are indicated.
