# Supplementary material for: Altered high-density lipoprotein composition and functions during severe COVID-19
Source: Sci Rep. 2021 Jan 27;11:2291. doi: 10.1038/s41598-021-81638-1 (PMC7841145; doi:10.1038/s41598-021-81638-1)

Altered high-density lipoprotein composition and functions during severe COVID-19

Floran Begue[1], Sébastien Tanaka[1,2], Zarouki Mouktadi[1], Philippe Rondeau[1], Bryan Veeren[1], Nicolas Diotel[1], Alexy Tran-Dinh[2,3], Tiphaine Robert[4], Erick Vélia[5], Patrick Mavingui[6], Marie Lagrange-Xélot[7], Philippe Montravers[2,3,8], David Couret[1,9,+] and Olivier Meilhac[1,10,*,+]

[1] Université de La Réunion, INSERM, UMR 1188 Diabète athérothombose Réunion Océan Indien (DéTROI), Saint-Denis de La Réunion, France.

[2]AP-HP, Service d'Anesthésie-Réanimation, CHU Bichat-Claude Bernard, 75018 Paris, France.

[3]Université de Paris, UFR Denis Diderot, Paris

[4]AP-HP, Service de Biochimie, CHU Bichat-Claude Bernard, 75018 Paris, France.

[5] Clinique Sainte-Clotilde, Groupe Clinifutur, Pôle mère enfant, 97490 Sainte-Clotilde, La Réunion, France.

[6] Université de La Réunion, UMR Processus Infectieux en Milieu Insulaire Tropical (PIMIT), INSERM 1187, CNRS 9192, IRD 249, 2 rue Maxime Rivière (GIP CYROI), 97490 Sainte-Clotilde, La Réunion, France.

[7] CHU de La Réunion, Service des maladies infectieuses, Saint-Denis, France.

[8] Inserm UMR 1152 Physiopathologie et épidémiologie des maladies respiratoires, Université Diderot, Paris

[9] CHU de La Réunion, Service de neuro-réanimation, Saint-Pierre, France.

[10] CHU de La Réunion, CIC-EC 1410, Saint-Pierre, France.

* Corresponding author: olivier.meilhac@inserm.fr

[+]These authors contributed equally to this work

Running Title: HDL in COVID-19

Figure 3A (upper panel) – anti SAA-1 antibody

as shown in the manuscript

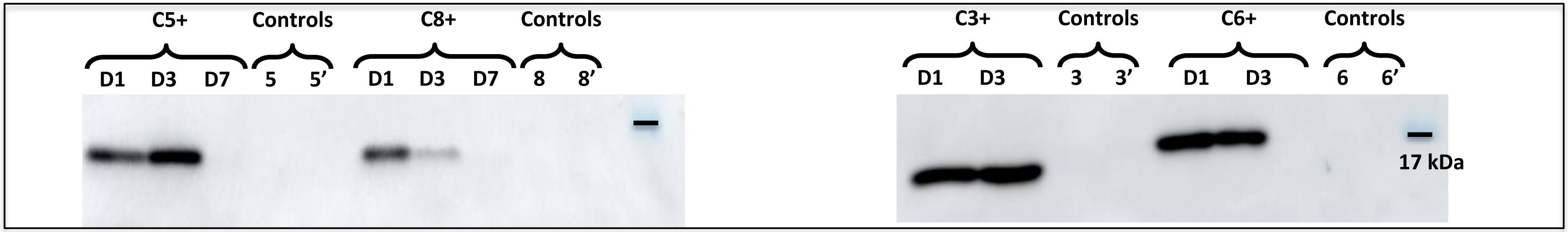

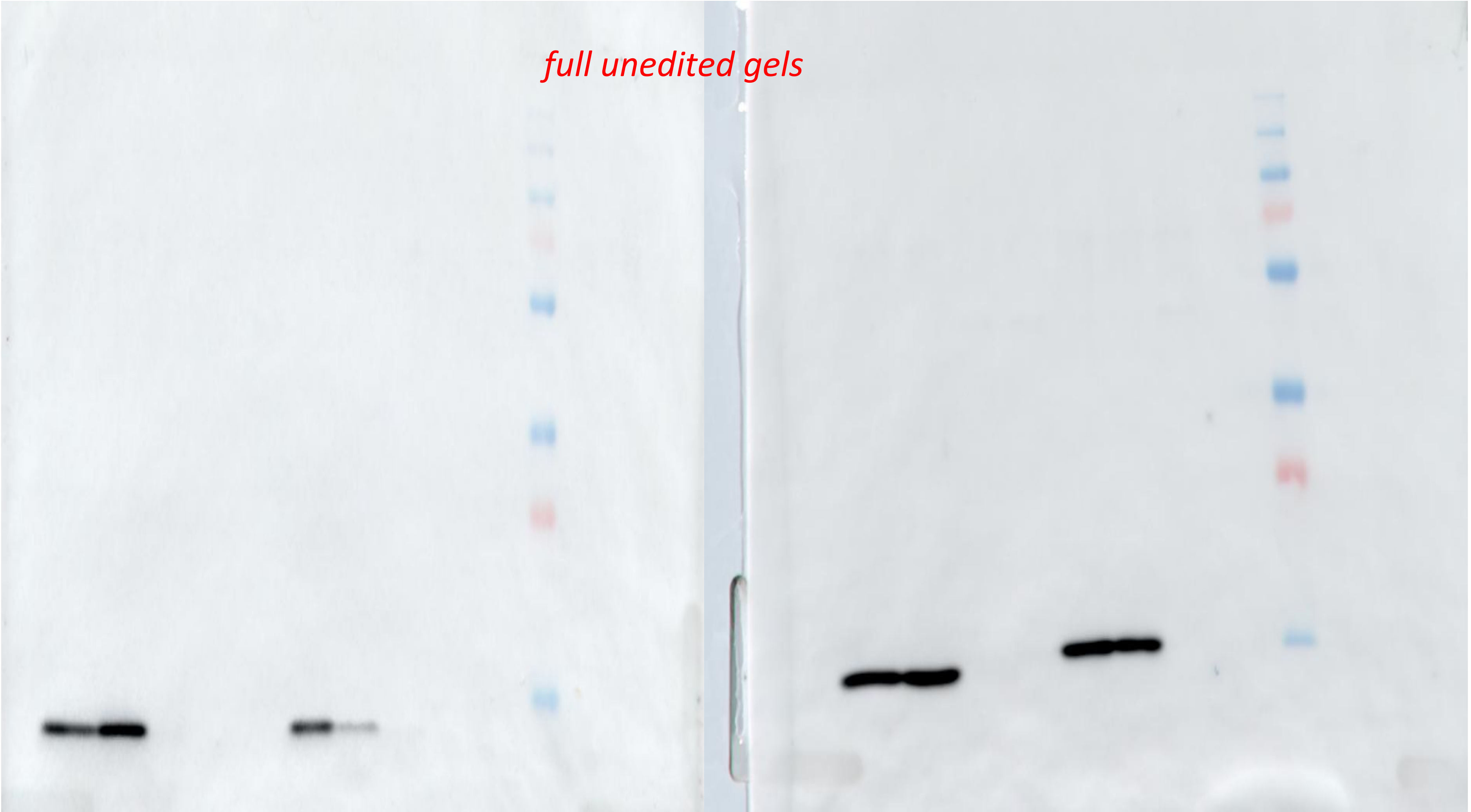

Figure 3A (lower panel) – anti SAA-1 antibody

as shown in the manuscript

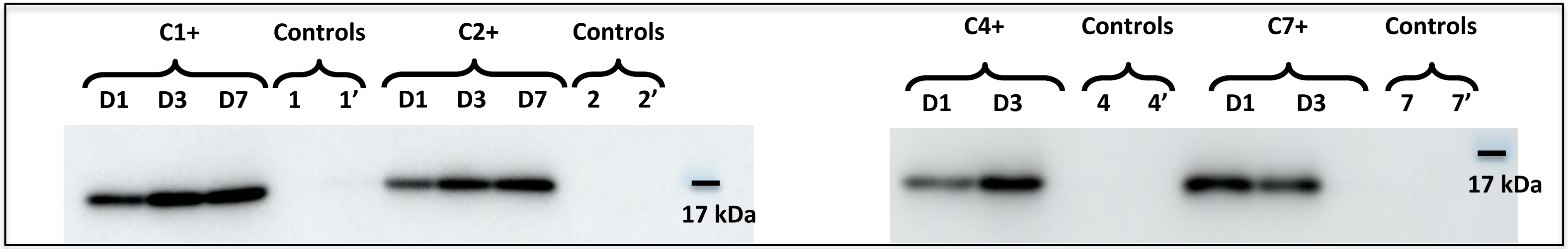

full unedited gels

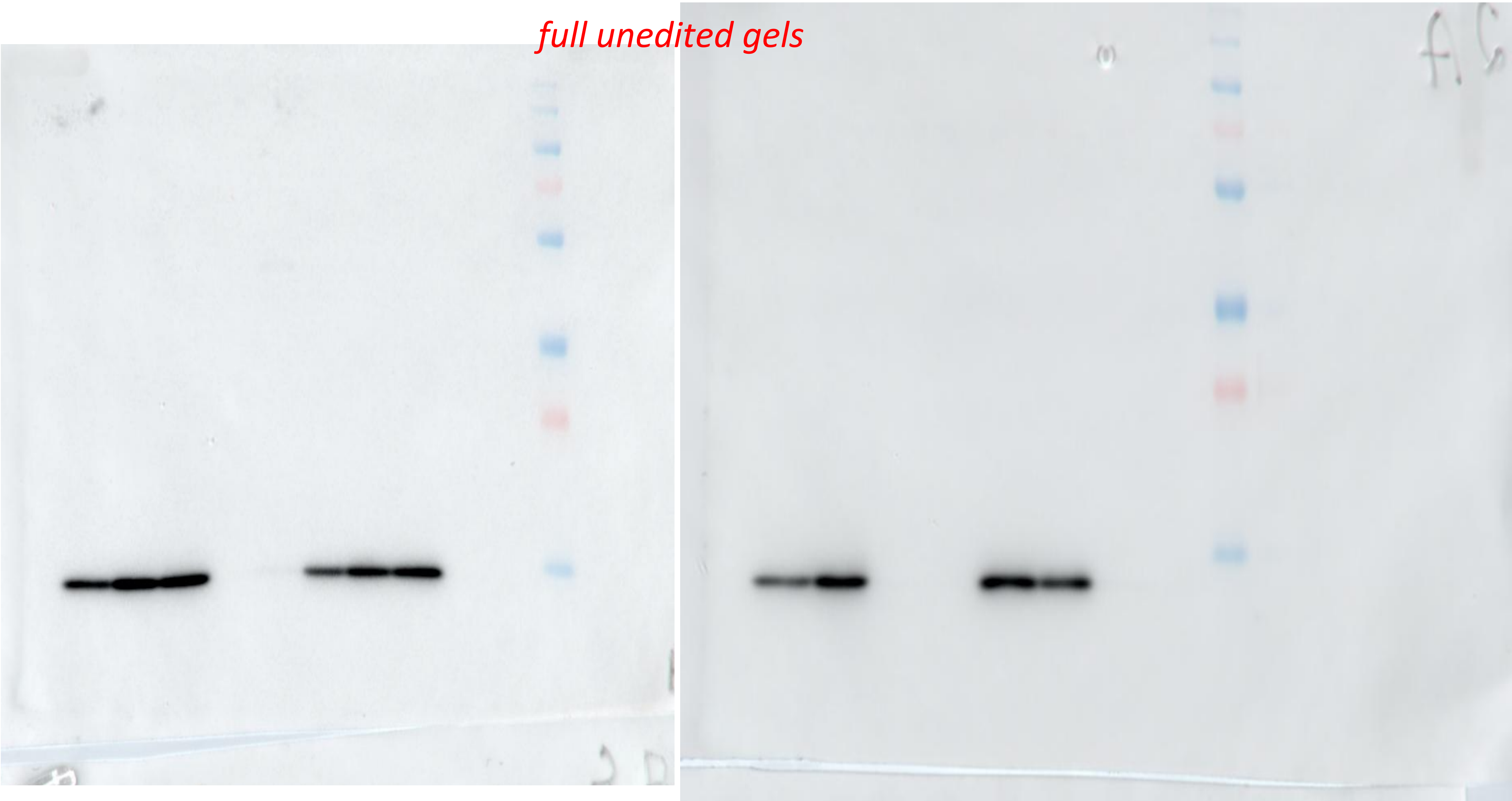

Figure 3B – anti PON-1 antibody

as shown in the manuscript

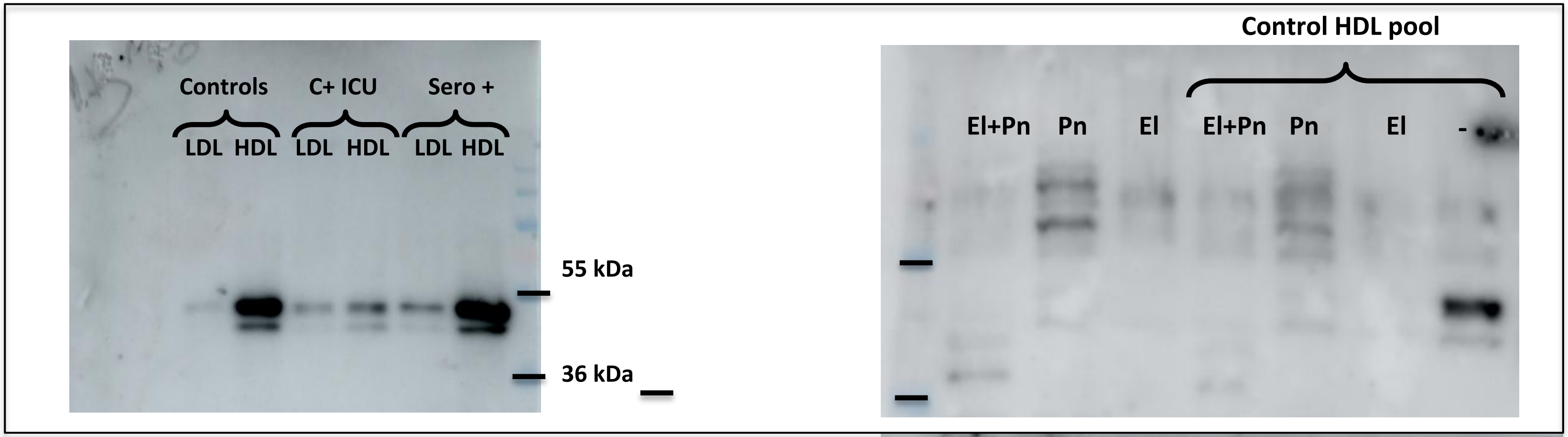

full unedited gels

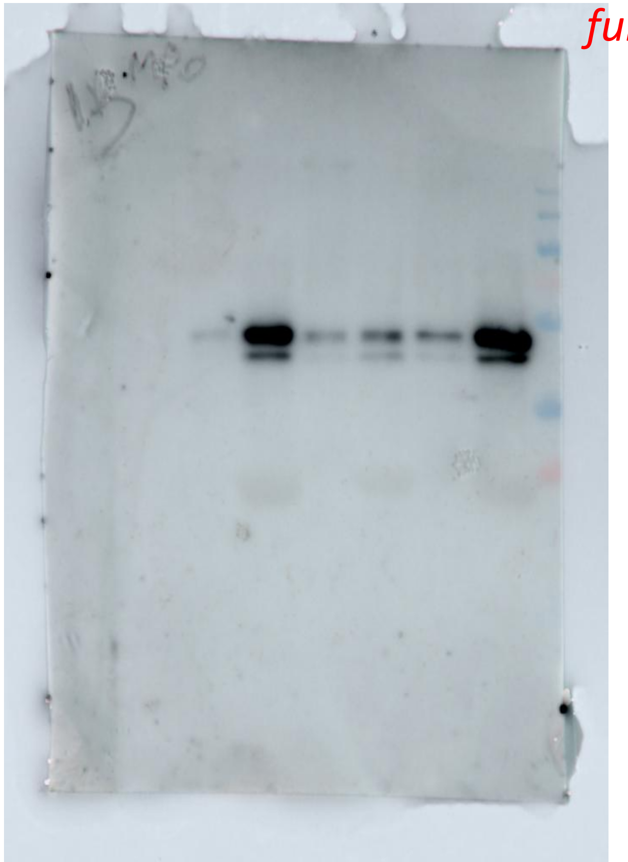

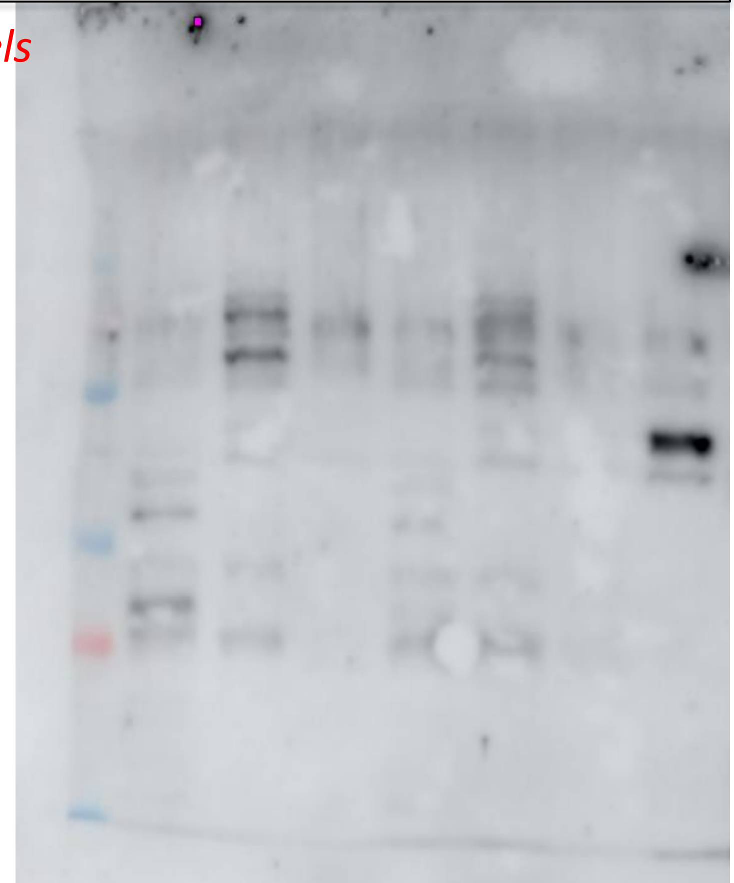

Supplemental figure 2– anti AATantibody

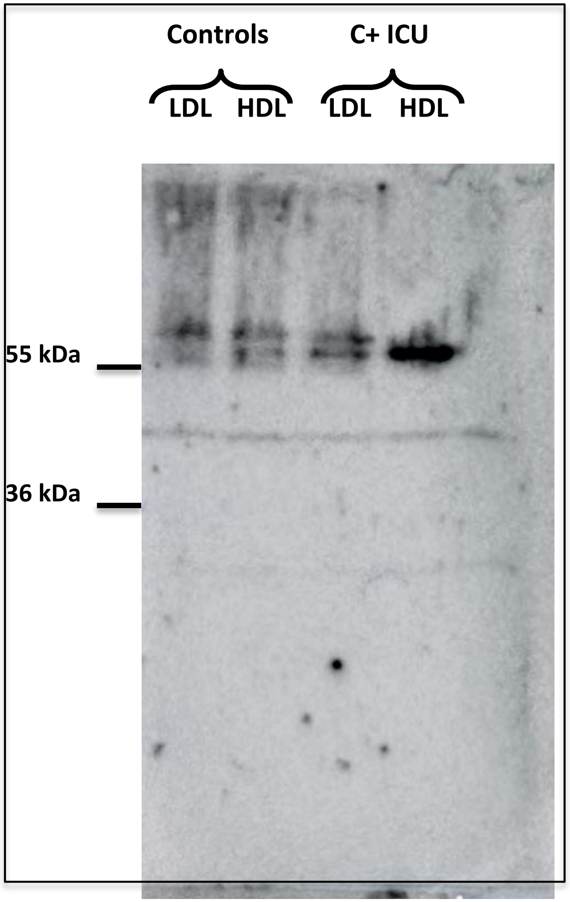

as shown in the manuscript

full unedited gel

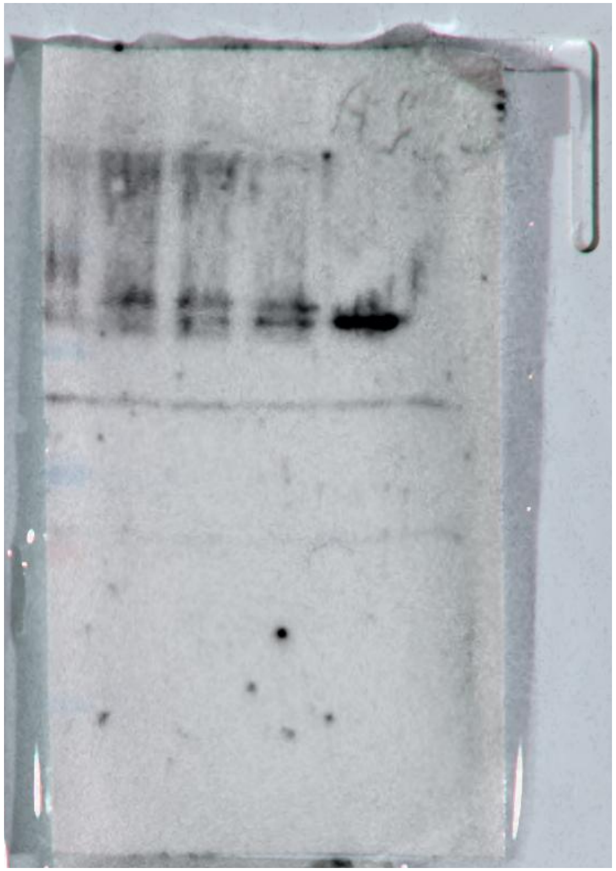

Supplement: Supplementary file 4 — Supplementary Information 4. [file 41598_2021_81638_MOESM4_ESM.pdf]
